# M(o)TOR of aging: MTOR as a universal molecular hypothalamus

**DOI:** 10.18632/aging.100580

**Published:** 2013-07-16

**Authors:** Mikhail V. Blagosklonny

**Affiliations:** Department of Cell Stress Biology, Roswell Park Cancer Institute, BLSC, L3-312, Elm and Carlton Streets, Buffalo, NY, 14263, USA

**Keywords:** senescence, geroconversion, rapamycin, diseases, molecular hypothalamus

## Abstract

A recent ground-breaking publication described hypothalamus-driven programmatic aging. As a Russian proverb goes “everything new is well-forgotten old”. In 1958, Dilman proposed that aging and its related diseases are programmed by the hypothalamus. This theory, supported by beautiful experiments, remained unnoticed just to be re-discovered recently. Yet, it does not explain all manifestations of aging. And would organism age without hypothalamus? Do sensing pathways such as MTOR (mechanistic Target of Rapamycin) and IKK-beta play a role of a “molecular hypothalamus” in every cell? Are hypothalamus-driven alterations simply a part of quasi-programmed aging manifested by hyperfunction and secondary signal-resistance? Here are some answers.

## New discovery

The secret of aging has been finally revealed [1]. As described by Cai and coworkers [2], the activity of the inflammatory pathway is increased with age in the hypothalamus, a place in the brain that connects sensory inputs of the nervous system with endocrine function [2, 3]. This small neuroendocrine region consists of neurons and glia cells and governs growth, metabolism and reproduction. With age, overactivated glia secrete proinflammatory cytokines, activating the IKK-beta/NF-kB pathway in neurons, leading to organismal aging [2-4]. Also, it was suggested that hypothalamic SIRT1 may regulate aging [5].

## Well-forgotten Old

In the paper entitled “On the … Elevation of the Activity of Certain Hypothalamic Centers” (Dilman, 1958), Vladimir Dilman suggested that aging is caused by a progressive loss of sensitivity of the hypothalamus coupled with increased activity of its certain centers. This caused progressive alteration of homeostasis, metabolic disturbances, leading to age-related diseases [6-13]. In other words, there is an age-related loss of sensitivity by the hypothalamus to the negative feedback of certain hormones, such as estrogens and glucocorticoids [6-13]. This explains the development of age-related diseases, including metabolic disorders and menopause. Furthermore, several agents including phenformin [11, 13] and its analog metformin extended life span in rodents [14-18]. Unfortunately, the hypothalamic theory was far ahead of its time and too medical to be appealing to gerontologists. Also, it cannot clearly link cellular and organismal aging and diseases. On one hand, aging (as a process) was assumed to be caused by accumulation of molecular damage. On the other hand, *manifestations* of aging such as age-related diseases were described as driven by the hypothalamic activity. Finally, it seemed odd that such a universal process as aging has such a specific driver, making it difficult to extend the theory to invertebrates (at that time).

## MTOR in geroconversion

**Figure 1:**
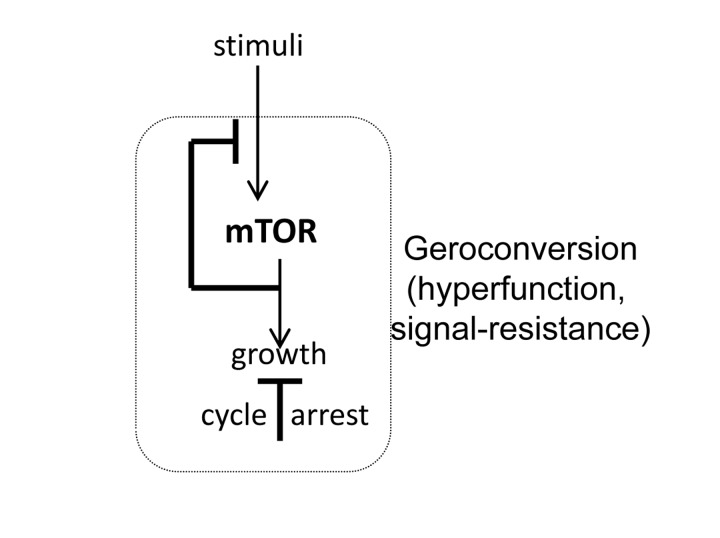
MTOR-dependent geroconversion to senescence When cell cycle is arrested, MTOR drives geroconversion, leading to cellular hypertrophy, hyperfunction, loss replicative/regenerative potential and resistance to signals such as insulin.

Meanwhile, in the field of cancer research, it was found that oncogenic/mitogenic signaling, such as Ras and Raf, can cause cellular senescence [19, 20]. Strong mitogenic signaling causes simultaneously (a) cell-cycle arrest and (b) hyper-activation of growth-promoting pathways, such as MEK/MAPK and MTOR (mechanistic target of rapamycin). The attention of investigators was attracted only to cell-cycle arrest because it is a barrier to cancer. Cellular senescence became almost a synonym of cell-cycle arrest. Yet, cell-cycle arrest is not senescence. It is growth-promoting pathways such as MTOR that cause the senescent phenotype (Fig. 1). When the cell cycle is arrested, still active MAPK and MTOR first force cell growth in size, followed by acquiring of hallmarks of senescence [21], a process that was named gerogenic conversion or geroconversion [22]. A senescent phenotype is characterized by hyperfunction (such as a hypersecretory and pro-inflammatory phenotype) and secondary signal resistance [21, 22]. Thus, pro-inflammatory and hypersecretory phenotypes are hallmarks of senescence [23-32]. Some other examples are contraction of smooth muscle cells, adhesion of platelets and bone resorption by osteoclasts, which lead to hypertension, thrombosis, and osteoporosis. Inhibition of MTOR suppresses geroconversion, preventing senescence, while maintaining cell cycle arrest [33-44]. Thus senescence is a continuation of cellular growth, when actual growth is constrained [33, 45, 46]. The MTOR is the primary cause of cellular aging, or senescence, leading to the well recognized diseases of aging [47-50].

## MTOR-driven quasi-programmed aging

As cellular aging is a continuation of growth, similarly, organismal aging is a continuation of organismal growth. Aging is not programmed but quasi-programmed, an aimless continuation of organism growth [47, 51-55]. Since geroconversion is driven by in part by MTOR, it was predicted that rapamycin should prolong life span in mammals [47, 56]. In fact, fibroblasts from long-lived mutant mice exhibit lower TOR activity [57] and rapamycin extends life span in mice [58-65]. It also inhibits chronological senescence, which is self-poisoning by acetic and lactic acids in yeast [66, 67] and mammalian cells [68, 69], respectively. Inhibition of MTOR also extends replicative lifespan in yeast [66, 70, 71] and mammalian cells [72, 73].

## MTOR as hypothalamus

Discovered by Michael Hall and co-workers in yeast [74], TOR (target of rapamycin) is a “molecular hypothalamus”. It integrates signals generated by insulin, mitogens, cytokines, oxygen, and nutrients [75-79]. Noteworthy, IKK-beta activates MTOR [80-83]. Given that IKK-beta is activated in aging hypothalamus, one may suggest that MTOR is activated too. In agreement, there is an age-dependent increase of MTOR signaling in hypothalamic neurons that express pro-opiomelanocortin. Systemic or intracerebral administra-tion of rapamycin causes weight loss in old mice [84].

## Sensing and aging in invertebrates

Although the hypothalamus as an organ is absent in worm, single neurons are analogs of the hypothalamus. *C. elegans* senses environmental signals through ciliated sensory neurons and mutations that cause defects in sensory cilia extend lifespan [85]. To a great extend, life span of *C. elegans* is regulated by environmental cues [86-88]. However, there are other determinants of *C. elegan* lifespan. For example, signals from the reproductive system regulate the lifespan of *C. elegans* and germline removal extends lifespan [89, 90].

## Hypothalamus is not (absolutely) necessary for aging

**Figure 2:**
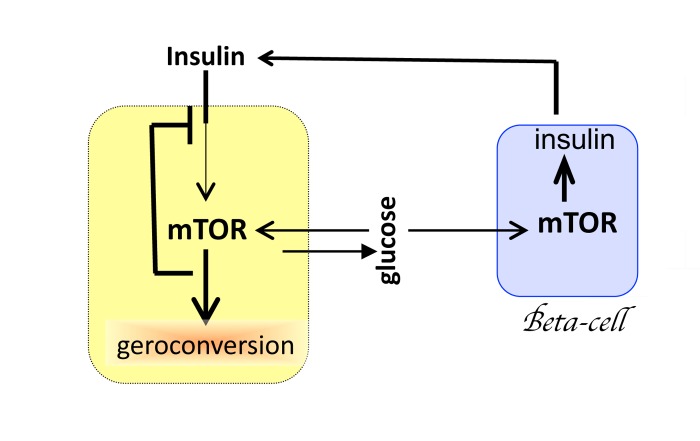
Mutual overstimulation of MTOR in beta-cells and insulin-dependent tissues Mutual and reciprocal over-stimulation leads to cellular hyperfunctions and secondary signal-resistance. By negative feedback, overactivated MTOR blocks insulin signaling. See text for explanation. Yellow cell: hepotocyte, adipocyte or muscle cell. Blue cell: beta-cell of the pancreas.

Despite its small size, the hypothalamus plays a disproportionally important role in aging. The reason is that the hypothalamus interacts with numerous organs and tissues, driving alterations of homeostasis. But aging would unfold without the hypothalamus. Mutual cell-cell stimulation, with positive feedback loops, causes overstimulation and geroconversion, manifested as hyperfunction and secondary signal-resistance. For example, glucose activates MTOR, causing resistance to insulin and IGF-1 (Fig. 2). In turn, activation of MTOR increases insulin production by beta-cells of the pancreas (Fig. 2). Increased production of, insulin further stimulates MTOR in fat, muscle and liver tissues, further promoting insulin-resistance. Insulin resistance increases levels of glucose, which over stimulates beta-cells to induce insulin and simultaneously making beta-cells resistant to survival signals (Fig. 2). The process may culminate in beta-cells failure and diabetes type II. This in turn will lead to renal failure, blindness and death. And the hypothalamus is not required (Fig. 2) because each cell contains a molecular “hypothalamus”.
